# An Additional 30-s Observation of the Right-Sided Colon Using a Novel Endoscopic System with Texture and Color Enhancement Imaging Decreases Polyp Miss Rates: A Multicenter Study

**DOI:** 10.3390/diagnostics15141759

**Published:** 2025-07-11

**Authors:** Yoshikazu Inagaki, Naohisa Yoshida, Hikaru Hashimoto, Yutaka Inada, Takaaki Murakami, Takahito Shimomura, Kyoichi Kassai, Yuri Tomita, Reo Kobayashi, Ken Inoue, Ryohei Hirose, Osamu Dohi, Yoshito Itoh

**Affiliations:** 1 Department of Gastroenterology, Nishijin Hospital, Kyoto 6028319, Japan; y.inaina@s7.dion.ne.jp (Y.I.); takashimomura8897@gmail.com (T.S.); kassai@nisijin.net (K.K.); 2 Department of Molecular Gastroenterology and Hepatology, Kyoto Prefectural University of Medicine, Kyoto 6028566, Japan; reipanna@koto.kpu-m.ac.jp (R.K.); keninoue71@koto.kpu-m.ac.jp (K.I.); osamu-d@koto.kpu-m.ac.jp (O.D.); yitoh@koto.kpu-m.ac.jp (Y.I.); 3 Department of Gastroenterology, Osaka General Hospital of West Japan Railway Company, Osaka 5450053, Japan; h-hashi@koto.kpu-m.ac.jp; 4 Department of Gastroenterology, Japanese Red Cross Kyoto Daiichi Hospital, Kyoto 6008811, Japan; yutak-i@koto.kpu-m.ac.jp; 5 Department of Gastroenterology, Aiseikai Yamashina Hospital, Kyoto 6078454, Japan; t-mura46@koto.kpu-m.ac.jp; 6 Department of Gastroenterology, Tomita Hospital, Kyoto 6168355, Japan; yuri0428@koto.kpu-m.ac.jp; 7 Department of Infectious Disease, Kyoto Prefectural University of Medicine, Kyoto 6028566, Japan; ryo-hiro@koto.kpu-m.ac.jp

**Keywords:** TXI, ADR, polyp miss, right-sided colon, colonoscopy

## Abstract

**Background/Objectives:** White light imaging (WLI) of colonoscopy has a 26% adenoma miss rate. We aimed to evaluate the effectiveness of an additional 30 s (Add-30s) observation of the right-sided colon using a novel system (EVIS X1; Olympus Co.) with texture and color enhancement imaging (TXI). **Methods:** We reviewed 515 patients who underwent colonoscopy with Add-30s TXI between February 2021 and December 2023 at three affiliated hospitals. After initial right-sided colon observation with WLI, the colonoscope was reinserted into the cecum, and the right-sided colon was re-observed with Add-30s TXI. Adenoma and sessile serrated lesion (SSL) detection rate (ASDR) and adenoma detection rate (ADR) were examined. Multivariate analysis identified factors influencing lesion detection using the Add-30s TXI. The difference in WLI and TXI between the novel and previous scopes was performed using propensity score matching (PSM). The efficacy of WLI with the novel system was compared to that of the previous system. **Results:** Among the 515 cases, Add-30s TXI observation increased right-sided ADR and ASDR by 7.4% and 9.5%, respectively. The multivariate analysis showed novel scope as an independent factor for adenoma and SSL detection (odds ratio: 2.41, *p* < 0.01). Right-sided ADR and ASDR for Add-30s TXI were significantly higher in the novel scope than the previous scope (ADR, 25.2% vs. 15.3%; *p* = 0.04; ASDR, 32.4% vs. 18.9%; *p* = 0.02). ASDR for WLI observation was significantly higher in the novel system than the previous system (34.8% vs. 25.9%; *p* < 0.01). **Conclusions:** Add-30s TXI significantly improved the detection of missed adenomas and SSLs in the right-sided colon.

## 1. Introduction

The removal of colorectal adenomas reduces the incidence of colorectal cancer (CRC) and CRC-related mortality [1,2,3]. Colonoscopy is an important tool for detecting colorectal adenomas. However, a systematic review reported a 26% miss rate for adenomas when using white light imaging (WLI) (95% confidence interval [CI]: 23–30%) [4]. Poor bowel preparation, right-sided location, flat morphology, small polyp size, and a high prevalence of sessile serrated lesions (SSLs) have been identified as risk factors for missed polyps [4,5,6,7,8,9].

Numerous clinical studies have reported improvements in adenoma detection rates (ADRs) with image-enhanced endoscopy, including narrow-band imaging (NBI), blue laser imaging, and linked-color imaging [10,11,12]. A recent systematic review demonstrated that NBI detected significantly more adenomas than WLI under optimal bowel preparation conditions [13]. In July 2020, a novel endoscopic system incorporating five-color light-emitting diodes (LEDs) (EVIS X1: CV-1500; Olympus Co., Tokyo, Japan) was released globally. In addition to enhancements in WLI and NBI, this system introduced texture and color enhancement imaging (TXI) [14]. TXI is designed to improve lesion visibility by enhancing texture, brightness, and color contrast, making lesions appear redder and more conspicuous. Several studies have shown that TXI offers superior visibility compared to WLI [15,16]. Recently, a multicenter randomized controlled trial (RCT) demonstrated a positive impact of TXI vs. standard high-definition WLI on ADR [17].

Additional observation of the right-sided colon has been shown to reduce missed polyps. A systematic review showed that supplementary forward and retroflexed observations of the right colon after the initial WLI observation increased right-sided ADR by 10% and 6%, respectively [18]. In a previous study, we reported the efficacy of an additional 30 s (Add-30s) NBI observation following standard WLI for reducing missed polyps in the right colon [19]. Notably, in a multicenter RCT, we demonstrated that Add-30s observation of the right-sided colon using TXI was non-inferior to NBI for detecting missed polyps, with both approaches improving ADR by 10.2% and 10.5%, respectively [20].

In the present multicenter study, we aimed to evaluate the effectiveness of Add-30s TXI observation of the right-sided colon in a large cohort. We also assessed the efficacy of the novel endoscopic system in this context.

## 2. Materials and Methods

**Figure 1:**
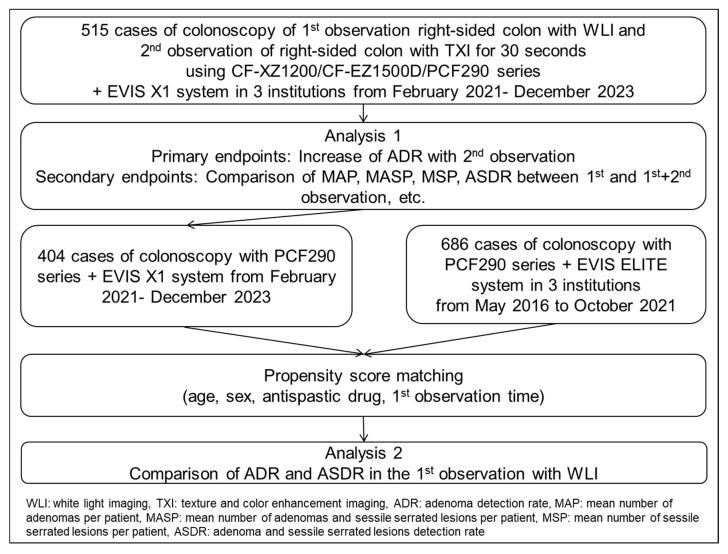
Study flowchart.

This multicenter, retrospective observational study was conducted at three affiliated hospitals in Japan: Nishijin Hospital, Kyoto Prefectural University of Medicine, and the Japanese Red Cross Kyoto Daiichi Hospital. We analyzed 515 patients who underwent Add-30s observation with TXI as a second observation following WLI in the right-sided colon between February 2021 and December 2023. Since we previously reported the effectiveness of Add-30s observation, this method was employed at the discretion of the endoscopist at each institution (Figure 1) [19]. All procedures were performed using either a 290-series scope (PCF-H290AZI, CF-HQ290ZL/I; Olympus Co., Tokyo, Japan) or a novel scope (CF-XZ1200L/I, CF-EZ1500DL/I; Olympus Co., Tokyo, Japan) in combination with the novel system (CV-1500; Olympus Co., Tokyo, Japan).

The inclusion criteria were as follows: (1) symptoms such as abdominal pain, constipation, anemia, and hematochezia; (2) surveillance after resection of polyps or cancer; and (3) positive fecal occult blood. Exclusion criteria were as follows: confirmed recurrent lesions with scarring after previous endoscopic mucosal resection (EMR) or polypectomy, T1–T4 colorectal cancers, and prior surgical resection involving the cecum or ascending colon. All examinations were performed by 14 endoscopists.

**Figure 2:**
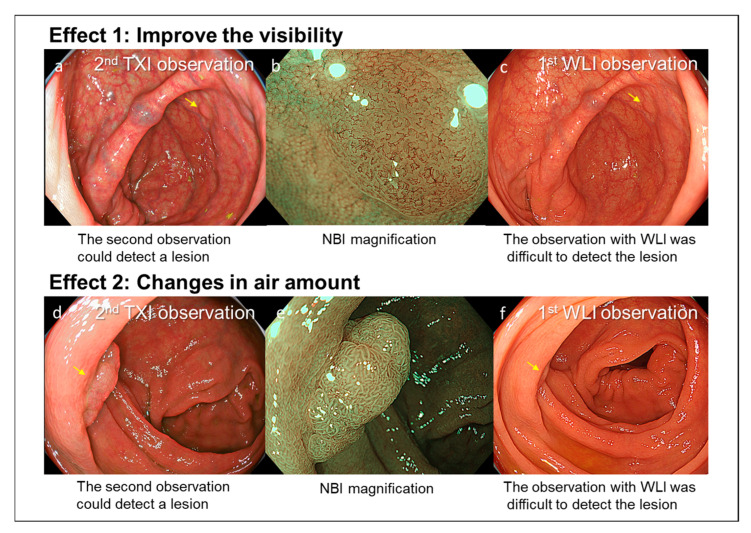
Case 1: A 2 mm non-polypoid polyp located in the cecum. (**a**) The lesion was detected during the second observation using TXI, which enhanced polyp visibility (yellow arrow). (**b**) NBI magnification findings were consistent with a typical adenoma. (**c**) A movie of the first observation was checked. The lesion was difficult to detect during the first WLI observation (yellow arrow). Case 2: A 10 mm polypoid polyp located in the ascending colon. (**d**) The lesion was detected during the second observation, adjusting the amount of insufflated air enabled visualization of a polyp hidden behind a mucosal fold (yellow arrow). (**e**) NBI magnification findings were consistent with a typical adenoma. (**f**) A movie of the first observation was checked. The lesion was difficult to detect during the first observation (yellow arrow).

Regarding the observation method, the cecum and ascending colon were first examined using WLI. Thereafter, the colonoscope was reinserted into the cecum from the hepatic flexure, and the right-sided colon, from the cecum to the ascending colon, was observed using Add-30s TXI (Figure 1). TXI1 was used, as in our previous study, because it provided better color enhancement than TXI2 and demonstrated superior visibility for colorectal lesions [20,21]. The Add-30s observation method involved sufficient insufflation of the right-sided colon to enable a distant view, following a previously reported protocol. The 30 s duration was based on our earlier study (Supplemental Video, Figure 2). Even if the right-sided colon was not completely observed during the 30 s, the second observation was nonetheless completed. Polyp location, morphology, and size were carefully documented to prevent duplicate polyp counts.

The primary endpoint of this study was the increase in ADR with the sAdd-30s TXI observation as the second observation. The secondary endpoints included the mean number of adenomas per patient (MAP), the mean number of adenomas and sessile serrated lesions per patient (MASP), the mean number of sessile serrated lesions per patient (MSP), and adenoma and SSL detection rates (ASDRs) between the first and combined first and second observations. Patient characteristics, such as age, sex, insertion time, bowel preparation, use of antispastic drugs, sedation, endoscopists, scope type, and duration of the first observation, and polyp characteristics, including size, location, morphology, and histology, were analyzed for both observations.

Additionally, these parameters were compared between the adenoma/SSL-detected and non-detected groups during the second observation using univariate and multivariate analyses. The ADRs and ASDRs from the first and second observations were also compared between groups that underwent colonoscopy using the novel and previous scopes. Using propensity score matching (PSM), we matched these two groups in a 1:1 ratio, selecting 111 patients in the novel scope group based on age, sex, and variables with *p*-values < 0.05 in the univariate analysis including the duration of the first observation time.

Finally, the ADR and ASDR of the first WLI observation using the novel system were compared with those of 686 colonoscopies performed between May 2016 and October 2021 using the previous system (EVIS LUCERA ELITE System, CV-190 [Video system]/CLV-290 [Light source], Olympus Medical Co., Tokyo, Japan) and 290-series scopes to determine whether the system affected detection outcomes (Figure 1). These two groups were also matched using PSM with 402 patients in the new system group at a 1:1 ratio, based on age, sex, and factors with *p* < 0.05 in univariate analysis (specifically, the use of antispastic drugs and the duration of the first observation).

To evaluate bowel preparation, the Aronchick bowel preparation score was used, which grades bowel preparation as excellent, good, fair, poor, or inadequate [22]. Excellent and good bowel preparation was defined as good in the present study. Midazolam was routinely administered for sedation. Endoscopic diagnosis of all lesions was performed using NBI magnification [23,24], and all polyps diagnosed as neoplastic or SSLs were resected using polypectomy, EMR, or endoscopic submucosal dissection (ESD) in accordance with the Japan Gastroenterological Endoscopy Society guidelines [25,26].

Some polyps that did not require endoscopic resection—such as hyperplastic or inflammatory polyps—were diagnosed based on magnified NBI observation rather than histological assessment due to the retrospective nature of the study. Experts were defined as endoscopists with experience performing more than 1000 colonoscopies and at least 50 withdrawal colonoscopies with TXI, according to previous reports [20].

Polyp size was defined by its maximum diameter and estimated based on comparison with snares or biopsy forceps. A cap was used for all procedures. Polyps were classified as polypoid or non-polypoid according to the Paris classification [27]. Histopathological diagnoses were made using biopsy or resected specimens in accordance with the World Health Organization classification. In this study, intramucosal cancer was categorized as high-grade dysplasia (HGD) [28]. SSLs were classified following the World Health Organization system [29].

Most patients consumed a liquid diet and 10 mL of sodium picosulfate the day before the colonoscopy. On the day of the procedure, they ingested 1.0–2.0 L of a highly concentrated polyethylene glycol solution with ascorbic acid (MoviPrep; EA Pharma Co., Ltd., Tokyo, Japan), followed by >0.5–1.0 L of water.

This retrospective study was conducted in accordance with the principles of the Declaration of Helsinki. After consultation with the Institutional Review Board (IRB) of each participating institution, the Ethics Committee of Kyoto Prefectural University of Medicine provided collective approval for the study (Approval No. ERB-C-1704-5, 5 June 2024). An opt-out of the study to the patients was performed in each hospital using a website or a board in an endoscopic unit, or both. Written informed consent was achieved from all the patients.

## 3. Statistical Analysis

The results were analyzed using the Mann–Whitney *U* test and the chi-squared test. Continuous variables, such as polyp size, were analyzed with the Mann–Whitney *U* test. Multivariate logistic regression analyses were also conducted to identify predictive factors for adenoma/SSL detection during the second observation. All statistical analyses were performed using SPSS software (version 22.0; IBM Japan, Tokyo, Japan). Statistical significance was defined as *p* < 0.05.

## 4. Results

Among the 515 cases, the mean age was 67.1 ± 11.0 years, and 60.6% were male (Table 1). The rate of good bowel preparation was 74.2%, and the use of the novel scope was observed in 21.6% of cases.

Regarding differences in detected lesions between the first WLI observation and the second Add-30s TXI observation, significant differences were noted in polyp size (mean ± SD: 4.8 ± 3.9 mm vs. 3.9 ± 3.9 mm, *p* < 0.01) and in the proportion of lesions located in the ascending colon (73.6% vs. 84.4%; *p* = 0.01) (Table 2).

Comparison of the first observation with the combined first and second observations showed significant increases in ADR (30.3% vs. 37.7%, *p* < 0.01) and ASDR (38.3% vs. 47.8%, *p* < 0.01) (Table 3). The increase in ADR was 7.4% (95% CI: 5.4–10.0). Significant differences were also observed in MAP, MASP, and MSP between the two observation strategies.

In the analysis of factors related to adenoma/SSL detection, only the use of the novel scope remained significant after PSM in the multivariate analysis (odds ratio: 2.41, 95% CI: 1.47–3.94; *p* < 0.01) (Table 4).

In the analysis of factors related to ADR, the use of the novel scope also remained significant after PSM in the multivariate analysis (odds ratio: 2.11, 95% CI: 1.25–3.57; *p* = 0.01) (Table 5).

Comparison between the novel and previous scopes showed that ADR and ASDR during the second Add-30s TXI observation were significantly higher in the novel scope group than in the previous scope group, according to PSM (ADR: 25.2% vs. 15.3%, *p* = 0.04; ASDR: 32.4% vs. 18.9%, *p* = 0.02) (Table 6).

Finally, with respect to the endoscopic system type, the ASDR during the first WLI observation was significantly higher in the novel system group than in the previous system group, based on PSM (34.8% vs. 25.9%; *p* < 0.01) (Table 7).

## 5. Discussion

In this multicenter study, we analyzed 515 patients who underwent Add-30s observation with TXI of the right-sided colon. This method increased the right-sided ADR by 7.4% and ASDR by 9.5%. We propose that the Add-30s TXI observation method has two main strengths (Figure 2). First, TXI enhances polyp visibility, thereby facilitating detection. Second, performing a second observation of the right-sided colon allows for the detection of polyps hidden behind mucosal folds, as the amount of insufflated air can be changed between observations.

A multicenter RCT reported that the ADR was 58.9% (221/375) in the TXI group and 42.7% (159/372) in the WLI group [risk ratio (RR): 1.38, 95% CI: 1.20–1.59; *p* < 0.001] [17]. Sakamoto et al. also reported in a multicenter study that TXI was a significant factor associated with MAP (OR: 1.4, 95% CI: 1.2–1.6; *p* < 0.001) and ADR (OR: 1.5, 95% CI: 1.0–2.3; *p* = 0.044), based on multivariate regression analysis [30]. On the other hand, a recent multicenter RCT from Japan reported that ADR was 57.2% and 56.0% in the TXI and WLI groups, respectively, with no statistically significant difference between the two groups. The polyp detection rate and flat polyp detection rate were significantly higher in the TXI group than in the WLI group, which were 82.5% vs. 74.4% (*p* = 0.003), and 76.5% vs. 70.3% (*p* = 0.036), respectively [31]. Further analysis is expected for the use of TXI as routine first observation. In the current study, consistent with these findings, TXI contributed to an increased ADR, although our study was a retrospective setting for improving polyp miss. Several studies have shown that colonoscopy is significantly less effective in reducing cancer incidence in the proximal colon compared to the distal colon [32,33]. Missed polyps in the proximal colon may account for this discrepancy, and TXI observation may help to mitigate this issue.

In the current study, polyps detected during the second Add-30s TXI observation were smaller and more frequently located in the ascending colon compared to those detected during the first WLI observation. In our previous RCT, Add-30s TXI observation of the right-sided colon achieved a significantly higher detection rate for polyps in the ascending colon compared to NBI (cecum/ascending colon: 14.3%/85.7% for TXI vs. 34.3%/65.7% for NBI; *p* < 0.01) [20]. Compared to NBI, TXI offers a brighter view and maintains adequate illumination even under suboptimal bowel preparation, making it potentially advantageous for detecting polyps in the ascending colon.

With WLI, additional observation of the right-sided colon can be performed using either the forward or retroflex view. A systematic review reported that additional forward and retroflex observations increased the right-sided ADR by 10% and 6%, respectively [18]. A recent RCT showed that the ADR for the right-sided colon in the second forward observation and first observation group was 27.1% and 21.6%, respectively (*p* = 0.042) [34]. Another meta-analysis found that the increase in right-sided colon ADR was lower in the retroflex view group than in the forward view group (8.1% vs. 11.3%; *p* = 0.04) [35]. However, additional WLI observation typically requires 2–3 min, and retroflex observation is technically challenging for both endoscopists and patients. In contrast, our Add-30s TXI observation achieved a comparable increase in ADR to additional WLI observations, making the short observation time the greatest advantage of this method.

The use of novel scopes resulted in higher ASDR and ADR during the second TXI observation compared to previous scopes. A previous multicenter study similarly demonstrated that the use of a new-generation scope was associated with an increased ADR [36]. The improved brightness and image quality of the novel scope, relative to previous models, may contribute to enhanced lesion detection, even when used with TXI. Furthermore, the new endoscopic system yielded a higher ASDR during WLI observation than the previous system. In our previous study using previous system, the MASP, MSP, and MAP for the first WLI observation of the right-sided colon were 0.43, 0.06, and 0.34, respectively [37]. In the current study using the novel system, the MASP, MSP, and MAP increased to 0.61, 0.14, and 0.50, respectively—higher than previously reported. To the best of our knowledge, system-based comparisons of this nature have not been previously reported; however, the improved resolution and brightness of the new system likely contributed to these findings.

This study has some limitations. First, it was a retrospective observational study. The Add-30s TXI observation primarily revealed minute polyps. There is potential for selection bias, as the cases included were not consecutive, and the decision to perform Add-30s TXI was made by the endoscopists at each hospital. Additionally, some cases included in this study overlapped with those in previous reports [20,37]. The comparison between the first observation and the combined first and second observations may also have been influenced by the polyp count during the first observation. The indication of the colonoscopy including positive fecal occult blood and family history were not examined in this study though they were likely to affect the ADR. Patient characteristics related to insulin resistance, including diabetes mellitus and body mass index, were not examined in this study, although these factors might be associated with right-sided polyps. Furthermore, approximately 20% of polyps were examined endoscopically but not histologically.

In conclusion, our study demonstrated that Add-30s TXI observation of the right-sided colon significantly improved ADR and ASDR. This method is simple to implement and effective in detecting missed polyps.

## Figures and Tables

**Table 1:** Clinical characteristics of patients.

Patient number	515
Age, years, mean ± SD	67.1 ± 11.0
Sex, % (*n*) male/female	60.6/39.4 (312/203)
Insertion time, s, mean ± SD	359 ± 203
Bowel preparation, % (*n*) excellent/good/fair/poor	20.2/54.0/23.9/1.9 (104/278/123/10)
Good preparation (excellent + good), % (*n*)	74.2 (382)
Antispastic drug, % (*n*)	75.7 (390)
Type of scope, % (*n*) novel scope/previous scope	21.6/78.4 (111/404)
Sedation, % (*n*)	26.6 (137)

SD: standard deviation.

**Table 2:** The results of the 1st observation and the 2nd observation.

	1st WLI	2nd TXI	*p* Value
Observation time for right-sided colon, s, mean ± SD	186 ± 117	30 ± 0	
Detected polyp number, *n*	326	141	-
Polyp size, mm, mean ± SD, (range)	4.8 ± 3.9 (2–30)	3.9 ± 3.9 (1–24)	<0.01
Polyp size, % (*n*) <5 mm/≥5 mm	63.2/36.8 (206/120)	75.2/24.8 (106/35)	0.01
Location, % (*n*) C/A	26.4/73.6 (86/240)	15.6/84.4 (22/119)	0.01
Morphology, % (*n*) polypoid/non-polypoid	65.6/34.4 (214/112)	67.4/32.6 (95/46)	0.75
Histopathology, % (*n*) HP/SSL/Ad	4.0/21.8/74.2 (13/71/242)	5.7/20.6/73.8 (8/29/104)	0.85
MAP, [95% CI] (*n*)	0.47 [0.43–0.51] (242)	0.20 [0,17–0.24] (104)	
MASP, [95% Cl] (*n*)	0.61 [0.56–0.65] (313)	0.26 [0.22–0.30] (133)	
MSP, [95% CI] (*n*)	0.14 [0.11–0.17] (71)	0.06 [0.04–0.08] (29)	

WLI: white light imaging; TXI: texture and color enhancement imaging; right-sided colon: cecum and ascending colon; SD: standard deviation; C: cecum; A: ascending colon; HP: hyperplastic polyp; SSL: sessile serrated lesion; Ad: adenoma; MAP: mean number of adenomas per patient; CI: confidence interval; MASP: mean number of adenomas and sessile serrated lesions per patient; MSP: mean number of sessile serrated lesions per patient.

**Table 3:** Comparison between 1st observation and 1st + 2nd observation.

	1st WLI	1st WLI + 2nd TXI	*p* Value
Right-sided ADR, % [95% CI] (*n*)	30.3 [26.5–34.4] (156)	37.7 [33.6–41.9] (194)	<0.01
Increase in ADR, % [95% CI]	-	7.4 [5.4–10.0]	-
Right-sided ASDR, % [95% CI] (*n*)	38.3 [34.2–42.5] (197)	47.8 [43.5–52.1] (246)	<0.01
Increase in ASDR, % [95% CI]	-	9.5 [7.3–12.4]	-
MAP, [95% CI] (*n*)	0.47 [0.43–0.51] (242)	0.67 [0.63–0.071] (346)	<0.01
MASP, [95% Cl] (*n*)	0.61 [0.56–0.65] (313)	0.87 [0.83–0.89] (446)	<0.01
MSP, [95% CI] (*n*)	0.14 [0.11–0.17] (71)	0.19 [0.16–0.23] (100)	<0.01

ADR: adenoma detection rate; CI: confidence interval; ASDR: adenoma + sessile serrated lesion detection rate; MAP: mean number of adenomas per patient, MASP: mean number of adenomas and sessile serrated lesions per patient; MSP: mean number of sessile serrated lesions per patient.

**Table 4:** Comparison of lesion-detected group and non-detection group in 2nd TXI observation.

	Univariate Analysis			Multivariate Analysis	
	Adenoma/SSL-Detected Group	Non-Detected Group	*p* Value	Odds Ratio (95% Cl)	*p* Value
Case number	104	411			
Age, years, mean ± SD	68.7 ± 10.9	66.7 ± 11.0	0.08	1.02 (0.99–1.04)	0.10
Sex, % (*n*), male/female	62.5/37.5 (65/39)	60.1/39.9 (247/164)	0.14	1.06 (0.65–1.74)	0.81
Bowel preparation, % (*n*), excellent/good/fair/poor	23.1/51.0/23.1/2.9 (24/53/24/3)	19.5/54.7/24.1/1.7 (80/225/99/7)	0.88		
Good preparation, (good, excellent), % (*n*)	74.0 (77)	74.2 (305)	0.80		
Antispastic drug, % (*n*)	76.9 (80)	75.4 (310)	0.43	1.28 (0.73–2.25)	0.40
Sedation, % (*n*)	32.7 (34)	25.1 (103)	0.07	1.53 (0.94–2.50)	0.08
Novel scope, % (*n*)	34.6 (36)	18.2 (75)	<0.01	2.41 (1.47–3.94)	<0.01
1st WLI observation time, s, mean ± SD	192 ± 118	184 ± 117	0.32	1.00 (0.99–1.00)	0.37

TXI: texture and color enhancement imaging; SSL: sessile serrated lesion; Cl: confidence interval; SD: standard deviation; WLI: white light imaging.

**Table 5:** Comparison of adenoma-detected group and non-detection group in 2nd TXI observation.

	Univariate Analysis			Multivariate Analysis	
	Adenoma-Detected Group	Non-Detected Group	*p* Value	Odds Ratio (95% Cl)	*p* Value
Case number	83	432			
Age, years, mean ± SD	69.1 ± 10.4	66.7 ± 11.1	0.29	1.03 (1.00–1.05)	0.10
Sex, % (*n*), male/female	68.7/31.3 (57/26)	59.0/41.0 (255/177)	0.11	1.65 (0.96–2.85)	0.07
Bowel preparation, % (*n*), excellent/good/fair/poor	25.3/50.6/21.7/2.4 (21/42/18/2)	19.2/54.6/24.3/1.9 (83/236/105/8)	0.61		
Good preparation, (good, excellent), % (*n*)	75.9 (63)	73.8 (319)	0.78		
Antispastic drug, % (*n*)	78.3 (65)	75.2 (325)	0.68	1.62 (0.86–3.07)	0.14
Sedation, % (*n*)	32.5 (27)	25.5 (110)	0.22	1.61 (0.94–2.77)	0.08
Novel scope, % (*n*)	34.9 (29)	19.0 (82)	<0.01	2.11 (1.25–3.57)	0.01
1st WLI observation time, s, mean ± SD	356 ± 224	359 ± 199	0.65	1.00 (0.99–1.00)	0.89

SSL: sessile serrated lesion, TXI: texture and color enhancement imaging, Cl: confidence interval, SD: standard deviation, WLI: white light imaging.

**Table 6:** Comparison of lesion detection according to scope type before and after propensity score matching.

	Pre Matching			After Matching		
Scope	Novel Scope	Previous Scope	*p* Value	Novel Scope	Previous Scope	*p* Value
Patient number, *n*	111	404		111	111	
Age, years, mean ± SD	68.4 ± 10.9	66.7 ± 10.0	0.19	68.4 ± 10.9	69.4 ± 11.2	0.54
Sex, % (*n*) male/female	75.7/24.3 (84/27)	56.4/43.6 (228/176)	<0.01	75.7/24.3 (84/27)	75.7/24.3 (84/27)	0.56
Antispastic drug, % (*n*)	75.7 (84)	75.7 (306)	1.00	75.7 (84)	67.6 (75)	0.12
1st WLI observation time, s, mean ± SD	169 ± 117	190 ± 117	0.04	169 ± 117	177 ± 122	0.33
1st WLI ADR, %, (*n*)	39.6 (44)	27.4 (111)	0.01	39.6 (44)	29.7 (33)	0.08
2nd TXI ADR, %, (*n*)	25.2 (28)	13.4 (54)	<0.01	25.2 (28)	15.3 (17)	0.04
1st WLI ASDR, %, (*n*)	49.5 (55)	35.1 (142)	<0.01	49.5 (55)	41.4 (46)	0.14
2nd TXI ASDR, %, (*n*)	32.4 (36)	16.8 (68)	<0.01	32.4 (36)	18.9 (21)	0.02

SD: standard deviation; WLI: white light imaging; ADR: adenoma detection rate; TXI: texture and color enhancement imaging; ASDR: adenoma + sessile serrated lesion detection rate.

**Table 7:** Comparison of lesion detection according to system (EVIS X1 vs. EVIS LUCERA ELITE) before and after propensity score matching.

	Pre Matching			After Matching		
System	Novel System	Previous System	*p* Value	Novel System	Previous System	*p* Value
Patient number, *n*	404	686		402	402	
Age, years, mean ± SD	66.7 ± 10.0	66.8 ± 11.9	0.65	66.7 ± 11.3	67.4 ± 11.9	0.39
Sex, % (*n*) male/female	56.4/43.6 (228/176)	384/304	0.46	56.5/43.5 (227/175)	58.5/41.5 (235/167)	0.62
Antispastic drug, % (*n*)	75.7 (306)	62.8 (431)	<0.01	76.1 (306)	76.1 (306)	1.00
1st WLI observation time, s, mean ± SD	190 ± 117	214 ± 123	<0.01	187 ± 121	192 ± 125	0.52
1st WLI ADR, %, (*n*)	27.4 (111)	25.8 (177)	0.30	27.4 (110)	22.6 (91)	0.14
1st WLI ASDR, %, (*n*)	35.1 (142)	29.8 (205)	0.04	34.8 (140)	25.9 (104)	<0.01

SD: standard deviation; WLI: white light imaging; ADR: adenoma detection rate; ASDR: adenoma + sessile serrated detection rate.

## Data Availability

The patient data used to support the findings of this study are available from the corresponding author upon request. However, identified patient data are restricted by the institutional review board of Kyoto Prefectural University of Medicine.

## Supplementary Materials

The following supporting information can be downloaded at: https://www.mdpi.com/article/10.3390/diagnostics15141759/s1, Supplemental Video: The cecum and ascending colon were first examined using WLI. Thereafter, the colonoscope was reinserted into the cecum, and the right-sided colon, from the cecum to the ascending colon, was observed using Add-30s TXI. The lesion was detected during the second observation using TXI, which enhanced polyp visibility.
